# Draft Genome Sequence of Pseudomonas simiae Strain 2-36, an *In Vitro* Antagonist of Rhizoctonia solani and Gaeumannomyces graminis

**DOI:** 10.1128/genomeA.01534-14

**Published:** 2015-02-05

**Authors:** Zaky Adam, Qing Chen, Renlin Xu, Adolf E. Diange, Eden S. P. Bromfield, James Tabi Tambong

**Affiliations:** Biodiversity, Environmental Health Program, Agriculture and Agri-Food Canada, Ottawa, Ontario, Canada

## Abstract

Pseudomonas simiae 2-36, isolated from a field plot under long-term mineral fertilization, exhibited strong *in vitro* antagonistic activities against Rhizoctonia solani and Gaeumannomyces graminis. We report here the draft genome sequence of Pseudomonas simiae 2-36, consisting of 6.4 Mbp with a 60.25% G+C content and 5,790 predicted protein-coding sequences.

## GENOME ANNOUNCEMENT

Pseudomonas simiae 2-36, isolated from a field plot under long-term mineral fertilization, exhibited strong *in vitro* antagonistic activities against Rhizoctonia solani and Gaeumannomyces graminis. The synthesis of molecules involved in antagonistic interactions and disease suppression is positively regulated by the *gacA* gene. A 500-bp *gacA* sequence derived from P. simiae 2-36 showed only 95.1% similarity to that of a known biocontrol agent, Pseudomonas sp. strain BSB1-2-37 (GenBank accession no. EF417461). Mutations in the *gacA*/*gacS* genes generally indicate phenotypic variations. We report here the draft genome sequence of P. simiae 2-36. The draft genome was determined by paired-end sequencing using Illumina MiSeq technology (at Génome-Québec, Montreal, Canada). A total of 7,902,862 paired-end reads, each 150 bp in length, totaling 1,185,429,300 bp, were obtained from 380-bp inserts. Quality checking using FastQC (http://www.bioinformatics.babraham.ac.uk/projects/fastqc/) showed that the reads were of sufficiently good quality such that no further trimming or error correction was required. The initial *de novo* assembly using ABySS version 1.5.2 (1) produced 54 contigs contained in 46 scaffolds, from which scaffolds with length <300 bp were removed. The remaining 21 scaffolds (minimum, 966 bp; maximum, 1,186,008 bp; *N*_50_, 755,011 bp; total size, 6,394,971 bp; total number of Ns, 1,715) were used for further analyses. SSPACE version 2.0 (2) was applied on the resulting scaffolds to extend and merge them into larger scaffolds based on read-pair information and short overlaps. This process reduced the number of scaffolds to 20 (minimum, 1,348 bp; maximum, 1,186,008 bp; *N*_50_, 755,011 bp; total size, 6,396,512 bp; total number of Ns, 1,716). GapFiller version 1.11 (3) was used to close the gaps between the short scaffolds that are contained within the 20 large scaffolds by replacing the unknown nucleotides (Ns) with true nucleotides based on read-pair information and short overlaps. The final draft genome consists of 20 scaffolds (minimum, 1,348 bp; maximum, 1,185,661 bp; *N*_50_, 754,763 bp) totaling 6,395,662 bp with 41 Ns. The G+C content of the draft genome is 60.25%, with an overall estimated coverage at 169×.

Mauve Contig Mover version 2.3.1 (4) was used to order the draft genome of P. simiae strain 2-36 using P. simiae WCS 417 (GenBank accession no. CP007637.1) as a reference genome. Automated annotation using the RAST annotation server (5) revealed that the draft genome of P. simiae 2-36 contains 5,790 predicted protein-coding sequences, of which 4,489 have assigned functions, 335 have proposed functions, 955 are considered hypothetical proteins, and 11 are proteins of unknown functions. The draft genome also contains 82 predicted noncoding RNAs, including 63 tRNAs, one pseudo-tRNA, and 18 rRNAs. The number of gene copies encoding 16S rRNA, 5S rRNA, and 23S rRNA are 6, 7, and 5, respectively. Compared to RAST, Glimmer version 3.02 (6), using open reading frames (ORFs) as a training set, predicted 5,811 genes, whereas RNAmmer version 1.2 (7) predicted 13 rRNAs containing 5, 7, and 1 copies of 16S rRNA, 5S rRNA, and 23S rRNA genes, respectively. The tRNAscan-SE version 1.3.1 software (8), however, predicted 65 tRNAs genes. Sixteen gene clusters involved in the synthesis of secondary metabolites, such as bacteriocin, nonribosomal peptides, and terpenes, were identified (9).

### Nucleotide sequence accession numbers.

This whole-genome shotgun project has been deposited at DDBJ/EMBL/GenBank under the accession no. JRMC00000000. The version described in this paper is the first version, JRMC01000000.
